# Efficacy of endoscopic ultrasound-guided versus surgical gastrojejunostomy for malignant gastric outlet obstruction: a systematic review and meta-analysis

**DOI:** 10.3389/fonc.2026.1943827

**Published:** 2026-10-09

**Authors:** Furui Zhong, Dali Xiong, Hao Zhang, Guosong Luo

**Affiliations:** 1 Department of General Surgery, Zigong Fourth People’s Hospital, Zigong, Sichuan, China; 2 2Department of Oncology Zigong, Fourth People’s Hospital, Zigong, Sichuan, China; 3 Department of Hepatobiliary and Pancreatic Surgery, Meishan People’s Hospital, Meishan, Sichuan, China; 4 Department of Center for Hepatobiliary-Pancreatic- Splenic Diseases Zigong Fourth People’s Hospital, Zigong, Sichuan, China

**Keywords:** endoscopic ultrasound-guided gastrojejunostomy, malignant gastric outlet obstruction, meta-analysis, surgical gastrojejunostomy, systematic review

## Abstract

**Objective:**

To systematically compare the key outcomes of endoscopic ultrasound-guided gastrojejunostomy (EUS-GJ) versus surgical gastrojejunostomy (SGJ) for the palliative treatment of malignant gastric outlet obstruction (MGOO).

**Methods:**

A systematic review and meta-analysis was conducted in accordance with the PRISMA statement. Databases including PubMed, Embase, Web of Science and Cochrane Library were comprehensively searched from inception to December 2025 for comparative studies of EUS-GJ and SGJ in treating MGOO. Meta-analysis was performed using a random-effects model, with subgroup and sensitivity analyses.

**Results:**

Eight studies involving 760 patients were included. Pooled analysis showed no significant differences between EUS-GJ and SGJ in technical success (RR = 0.98, P = 0.08), clinical success (RR = 1.08, P = 0.07), obstruction recurrence (RR = 0.48, P = 0.05), or re-intervention rates (RR = 1.48, P = 0.61). However, the EUS-GJ group demonstrated significant advantages, including a shorter length of hospital stay (MD= -3.57 days, P<0.001), a quicker time to first oral intake (MD= -2.88 days, P<0.001), a shorter time to resume chemotherapy (MD= -16.05 days, P<0.001), and a lower overall adverse events rate (RR = 0.44, P = 0.008).

**Conclusion:**

For the palliative management of MGOO, EUS-GJ showed no statistically significant difference in primary efficacy endpoints compared with SGJ, but it might be associated with faster recovery and improved safety.

**Systematic review registration:**

https://www.crd.york.ac.uk/PROSPERO/, identifier CRD420261308316.

## Introduction

Malignant gastric outlet obstruction (MGOO) is a severe complication of upper gastrointestinal malignancies such as advanced gastric cancer, pancreatic cancer, and duodenal cancer, with an incidence of up to 10%-25% (1–3). Its core pathological change is the obstruction of gastric content transit into the duodenum caused by tumor infiltration or compression, manifesting clinically as intractable nausea and vomiting, eating disorders, malnutrition, and rapid weight loss, which significantly impairs patients’ quality of life (4). Most patients are in the terminal stage of tumors, and the core of treatment is focused on palliative symptom relief, improvement of nutritional intake, and shortening of recovery time (5, 6).

Surgical gastrojejunostomy (SGJ) serves as a traditional palliative treatment strategy that restores enteral nutrition by establishing a bypass anastomosis between the stomach and jejunum, and has long been regarded as the gold standard for MGOO treatment (7, 8). However, SGJ, especially the open approach, has limitations including high invasiveness, long operative time, elevated incidence of postoperative complications (e.g., infection, bleeding, anastomotic leakage), prolonged hospital stays (9, 10). Moreover, MGOO patients often have cachexia and decreased multi-organ functional reserve, making some of them unable to tolerate the trauma of surgical operation (11). With the development of minimally invasive endoscopic technology, endoscopic ultrasound-guided gastrojejunostomy (EUS-GJ) has been gradually applied in clinical practice due to its advantages of minimal invasiveness, convenient operation and rapid recovery (12, 13). EUS-GJ establishes a gastrojejunal pathway under the guidance of endoscopic ultrasound using lumen-apposing metal stents (LAMS) and achieves bypass reconstruction without laparotomy, thus providing a new option for patients intolerant to surgery (14).

In recent years, numerous clinical studies have attempted to compare the efficacy and safety of EUS-GJ and SGJ, but the existing evidence has obvious heterogeneity. On the one hand, study designs include randomized controlled trials (RCTs) and retrospective cohort studies with a huge variation in sample size (10 to 20930 cases) (15, 16), and the results of some small-sample studies lack stability. On the other hand, surgical approaches have gradually evolved from traditional open surgery to minimally invasive laparoscopic and robotic surgery, and the differences in invasiveness and recovery speed among different approaches may affect the horizontal comparison of treatment outcomes (17). In addition, the definitions of core outcomes (e.g., clinical success rate, obstruction recurrence rate) have not been fully unified in existing studies, and some studies lack long-term follow-up data, leading to inconsistent conclusions among different studies. Some studies suggest that EUS-GJ has more advantages in safety and recovery speed, while others support that SGJ has better efficacy durability (18).

While previous systematic reviews and meta-analyses have established the efficacy and safety of EUS-GJ compared to SGJ for the MGOO, the evidence base continues to evolve with the publication of new, high-quality comparative studies. Early work by Bomman et al. (19) suggested equivalent clinical success and a lower adverse events rate for EUS-GJ, although their analysis was limited to six retrospective studies involving 484 patients and did not address key secondary endpoints such as length of hospital stay or functional recovery. Subsequent meta-analyses by Li et al. (20) and Miller et al. (17) provided broader evidence, further confirming the safety profile of EUS-GJ and its comparable clinical success to SGJ. However, these prior analyses predominantly included studies published before 2022, thereby lacking the most recent high-level evidence, particularly RCTs emerging since 2023. Furthermore, existing syntheses lack in-depth subgroup exploration regarding how the technical evolution of SGJ itself, such as the increasing adoption of minimally invasive approaches (laparoscopic/robotic), affects its comparative outcomes against EUS-GJ. Perhaps most critically, patient-centered recovery metrics crucial for quality of life and treatment continuity in this advanced cancer population, including time to oral intake and time to resumption of chemotherapy, have not been systematically evaluated in prior pooled analyses.

Given these evidence gaps, this study aims to conduct an updated systematic review and meta-analysis. In contrast to previous research, our analysis is the first to incorporate the most recent clinical studies up to 2025, including pivotal RCT data, increasing the number of included studies to eight with a total sample size of 760 patients, representing one of the largest direct comparative syntheses in this field to date. We not only re-evaluated traditional endpoints such as technical success, clinical success, adverse events, and reintervention rates but also placed emphasis on analyzing previously underreported indicators of rapid patient recovery, including the length of hospital stay, time to oral intake, and time to chemotherapy resumption. Additionally, within the meta-analytic framework, we explored the differential impact of minimally invasive (laparoscopic/robotic) versus traditional open surgical approaches within the SGJ cohort on clinical outcomes, a comparison that, to our knowledge, has rarely been systematically examined in prior meta-analyses. By providing this comprehensive assessment that integrates the latest evidence, a broader set of clinically relevant endpoints, and more nuanced subgroup analyses, we endeavor to offer a more robust and contemporary evidence base to inform optimal, individualized treatment selection for the palliative management of MGOO.

## Methods

### Protocol and registration

This study was strictly conducted in accordance with the Preferred Reporting Items for Systematic Reviews and Meta-Analyses (PRISMA) statement (21) and has been registered in the PROSPERO database (CRD420261308316). All analytical procedures were consistent with the predefined protocol without substantial modifications.

### Inclusion and exclusion criteria

The inclusion criteria were based on the predefined PICOS framework:

- Participants (P): Adult patients (aged ≥18 years) with MGOO confirmed by histology or imaging, caused by gastric cancer, pancreatic cancer, duodenal cancer, etc. Tumor stage was not restricted, but the study must be conducted in a palliative treatment setting.
- Intervention (I): EUS-GJ group, involving the creation of a gastrojejunal anastomosis using LAMS under EUS guidance.
- Comparison (C): SGJ group, including open, laparoscopic, or robotic approaches for creating a gastrojejunal anastomosis.
- Outcomes (O): The outcomes included technical success (successful creation of anastomosis), clinical success (resumption of oral intake with a Gastric Outlet Obstruction Scoring System [GOOSS] score ≥2), adverse events rate, length of hospital stay, as well as efficacy and prognostic indicators.
- Study design (S): RCT, prospective or retrospective cohort studies (sample size ≥ 10 patients).

Exclusion criteria: Studies with mixed patients with benign gastric outlet obstruction (e.g., peptic ulcer cicatricial stenosis, congenital malformations, chronic pancreatitis, etc.); non-comparative studies (reporting data of a single treatment group only), case reports, reviews, animal experiments and duplicate publications; studies with ambiguous definitions of outcome indicators or unavailable data extraction.

### Literature search strategy

A comprehensive literature search was performed in the PubMed, Embase, Web of Science and Cochrane Library databases from their inception to December 2025. Keywords were combined using Boolean logical operators, with the main terms including: “endoscopic ultrasound-guided gastroenterostomy”, “EUS-GJ”, “surgical gastrojejunostomy”, “SGJ”, “malignant gastric outlet obstruction”, “MGOO”, “gastric outlet obstruction” and their synonyms and MeSH terms. Detailed search strategies for each database are provided in **Supplementary Table S1**. The reference lists of the included studies were manually searched, and abstracts from international conferences such as Digestive Disease Week (DDW) and United European Gastroenterology Week (UEGW) (2020–2025) were also retrieved to ensure no omission of grey literature.

### Study selection and data extraction

Study selection was performed independently by two investigators. Disagreements were resolved through discussion or consultation with a third reviewer. After removing duplicates using EndNote software, two investigators independently screened titles and abstracts to exclude obviously irrelevant studies. Full texts of potentially eligible studies were obtained and assessed independently against the inclusion criteria by two reviewers.

Data were extracted independently by two investigators using a standardized form. Discrepancies were resolved by consensus with a third reviewer. Extracted data included: 1) Study characteristics: first author, publication year, study design, sample size, follow-up duration; 2) Baseline characteristics: patient age, gender, tumor type, SGJ procedure; 3) Outcome data: for dichotomous outcomes (e.g., technical success), event counts and total numbers were extracted; for continuous outcomes (e.g., hospital stay), means and standard deviations (SD) were extracted. If medians and ranges/interquartile ranges (IQR) were reported, they were converted to mean ± SD using the method proposed by Wan et al. (22). Operational definitions for adverse events, recurrence, reintervention, time to first oral intake, and time to resume chemotherapy for each included study are summarized in **Supplementary Table S2**.

### Risk-of-bias assessment

The Cochrane Risk of Bias (ROB 2) tool (23) was used to assess the risk of bias in RCTs, evaluating five domains: randomization process, deviations from intended interventions, missing outcome data, measurement of the outcome, and selection of the reported result. Each domain was judged as “low risk,” “some concerns,” or “high risk,” leading to an overall risk of bias judgment. The Risk of Bias in Non-randomized Studies of Interventions (ROBINS-I) tool (24) was used for non-randomized studies. It assesses bias across seven domains: confounding, Selection of participants, measurement classification of interventions, deviations from intended interventions, missing data, measurement of outcomes, and selection of the reported result. Each domain was rated as “low,” “moderate,” “serious,” or “critical” risk of bias, culminating in an overall judgment. Two reviewers independently assessed the risk of bias, and any disagreements were resolved through discussion or arbitration with a third reviewer.

### Quality of evidence assessment

We assessed the certainty of evidence for each outcome using the Grading of Recommendations Assessment, Development and Evaluation (GRADE) (25) approach. According to GRADE methodology, evidence from randomized controlled trials was initially rated as high certainty, while evidence from observational studies was initially rated as low certainty. The certainty rating for each outcome was then judged against five domains: risk of bias, inconsistency, indirectness, imprecision, and publication bias, and could be downgraded or upgraded accordingly. Two reviewers independently evaluated the certainty of evidence, and disagreements were resolved by consensus. The final certainty for each outcome was classified as high, moderate, low, or very low.

### Statistical analysis

Statistical analyses were performed using RevMan 5.4 and R software, with the significance level set at P<0.05. For dichotomous outcomes, risk ratios (RR) with 95% confidence intervals (CI) were calculated and pooled using the Mantel-Haenszel method. For continuous outcomes, mean differences (MD) with 95% CIs were calculated and pooled using the Inverse Variance method. Heterogeneity was assessed using the Q test (significance level α=0.10) and I^2^ statistic: I^2^<50% and P>0.10 indicated low heterogeneity; 50%≤I^2^<75% or P ≤ 0.10 indicated moderate heterogeneity; I^2^≥75% and P ≤ 0.10 indicated high heterogeneity. Subgroup analyses were conducted according to study design (RCT vs. N-RCT) and surgical approach (minimally invasive vs. open) to explore the sources of heterogeneity. Sensitivity analysis was performed by sequentially excluding a single study and re-pooling the effect sizes to observe the stability of the results. Egger’s test and visual inspection of funnel plots were used to assess publication bias.

## Results

### Literature search results

The initial search identified 468 records. After removing 182 duplicates, 286 records underwent title/abstract screening, of which 224 were excluded. The remaining 62 full-text articles were assessed for eligibility. After further full-text reading, 54 studies were excluded due to intervention inconsistency (n=19), inability to extract independent outcome data (n=8), duplicate publications or data overlap (n=10), defective study design (n=7), sample size less than 10 cases (n=6), and outcome data deficiency (n=4). Ultimately, 8 eligible studies (26–33) were included. The selection process is summarized in the PRISMA flow diagram (**Figure 1**).

**Figure 1:**
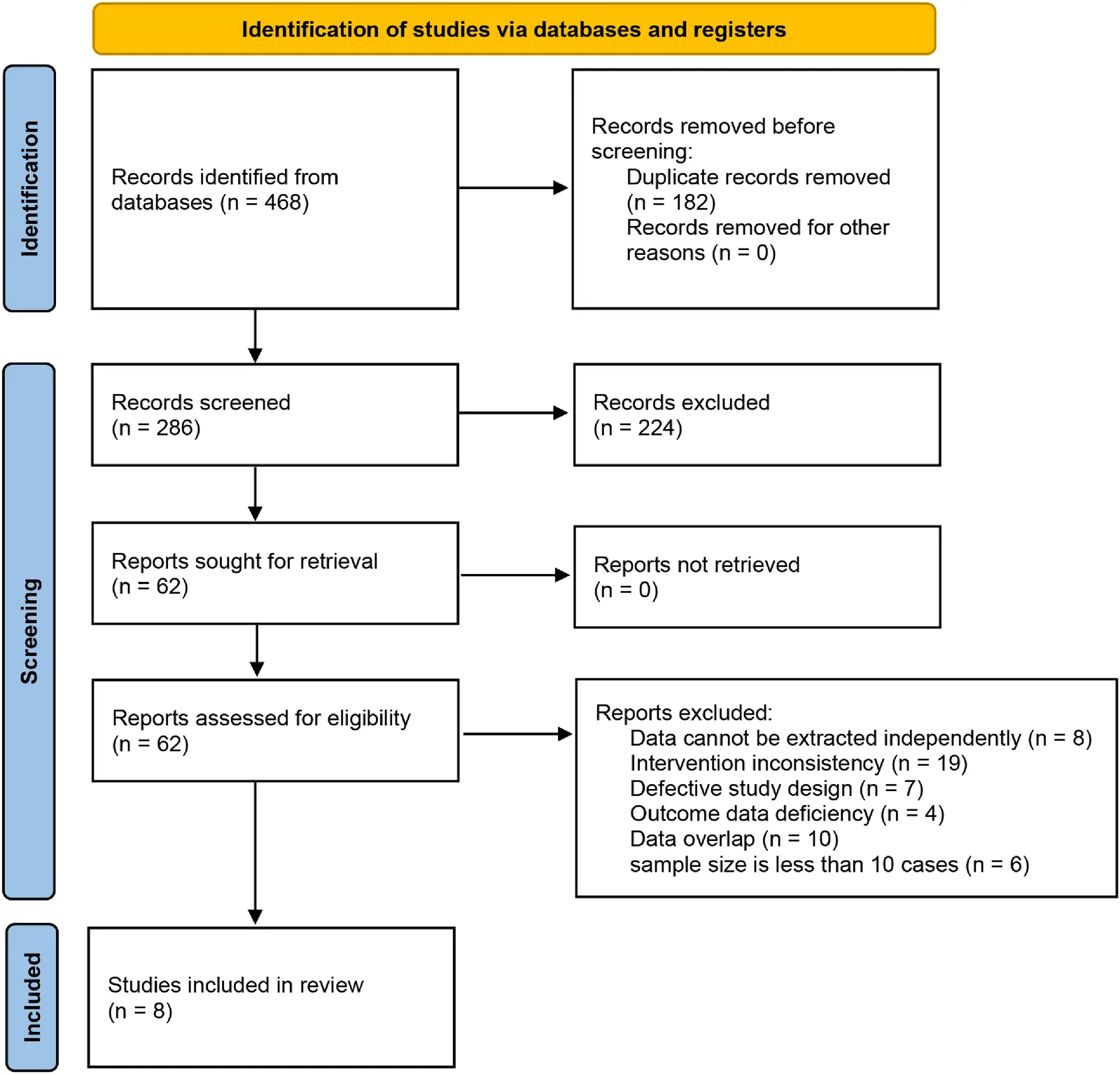
Flowchart of the systematic review process.

### Characteristics of included studies

The eight included studies comprised 2 multicenter RCTs (27, 31) and 6 retrospective cohort studies (26, 28–30, 32, 33), involving a total of 760 patients (397 in the EUS-GJ group and 363 in the SGJ group). The publication years ranged from 2018 to 2025, with individual study sample sizes per study ranging from 24 to 310. Pancreatic and gastric cancers were the predominant etiologies of obstruction. Follow-up durations varied considerably across studies, ranging from 6 days to 1274 days. The SGJ group included open, laparoscopic, and robot-assisted surgical approaches. Detailed baseline characteristics are presented in **Table 1**.

**Table 1:** Baseline characteristics of the included studies.

Author (Publication Year)	Research type	Included time period	Sample size		Age (years)		Sex (M/F)		Type of cancer (%)		Follow-up period (days)		SGJ procedure
			EUS-GJ	SGJ	EUS-GJ	SGJ	EUS-GJ	SGJ	EUS-GJ	SGJ	EUS-GJ	SGJ	
Khashab (29)	N-RCT	2013–2015 ^a^;2006–2011 ^b^	30	63	70 ± 13.3	68 ± 9.6	17/13	33/30	Pancreatic: 57%; Gastric: 17.6%; Ampullary: 6.7%; Others: 18.7%	Pancreatic: 84.5%; Ampullary: 14%; Others: 1.5%	115 ± 63	196 ± 155	OGJ
Widmer (32)	N-RCT	2015-2018	10	14	63 ± 13.5	68 ± 9.8	5/5	5/9	Pancreatic: 40%; Gastric: 20%; Duodenal: 20%; Colorectal: 10%	Pancreatic: 28.6%, Gastric: 7%, Duodenal: 21.4%, Others: 42.9%	90 (30-180)	240(30-35 1050)	OGJ: 78.6%; LGJ: 21.4%
Abbas (26)	N-RCT	2016-2020	25	27	65 ± 3.3	59 ± 4.8	11/14	13/14	Pancreatic: 40%, Cholangiocarcinoma: 20%, Others: 36%	Pancreatic: 33%; Gastric: 22%; Small intestine: 11%; Others: 34%	98	199	OGJ: 93%; LGJ: 7%
Chan (28)	N-RCT	2012-2020	30	35	64 ± 14	68 ± 10.5	22/8	23/12	Gastric: 26.7%, Pancreatic: 26.7%, Gallbladder: 10%, Others: 36.6%	Gastric cancer: 68.6%, Pancreatic cancer: 20%, Others: 11.4%	74 (6-703)	165 (6-1274)	LGJ
Canakis (3^33^)	N-RCT	2017-2022^a^; 2005-2020^b^	187	123	67.5 ± 12.6	61.6 ± 13.5	76/111	68/55	Pancreatic: 38.5% Gastric: 9.6%, Duodenal: 14.4% Metastatic: 21.4%	Pancreatic: 53.7% Gastric: 7.3%, Duodenal: 7.3% Metastatic: 5.6%	196	196	OGJ: 64%; LGJ: 36%
Pawa (30)	N-RCT	2018-2022^a^;2016-2022^b^	29	15	66.3 ± 12.6	64.1 ± 13.7	19/10	8/7	Pancreatic: 52%; Metastatic: 21%; Duodenal: 10%; Others: 17%	Pancreatic: 67%; Metastatic: 7%; Duodenal: 13%; Gastric: 13%	72	277	RGJ
Pavert (31)	RCT	2022-2024	48	50	69.4 ± 9.2	70.6 ± 10.5	28/20	27/23	Pancreatic:58%; Duodenal:8%; Cholangiocarcinoma:8%; Metastasis: 6%; Others: 20%	Pancreatic:50%; Duodenal:14%; Cholangiocarcinoma:12%; Metastasis: 6%; Others: 18%	168	168	LGJ
Bang (27)	RCT	2022-2024	38	36	65.3 ± 15.1	61.5 ± 12.3	23/15	21/15	Pancreaticobiliary: 57.9%; Gastroduodenal: 18.4%; Other: 23.7%	Pancreaticobiliary: 55.6%; Gastroduodenal: 36.1%; Other: 8.3%	180	180	LGJ or RGJ

RCT, randomized controlled trial; M/F, male/female; EUS-GJ, endoscopic ultrasound-guided gastrojejunostomy; SGJ, surgical gastrojejunostomy; OGJ, open gastrojejunostomy; LGJ, Laparoscopic gastrojejunostomy; RGJ, Robot-assisted gastrojejunostomy.

a the time period of the SGJ group.

b the time period of the EUS-GJ group.

### Risk of bias

Both included RCTs were at a low overall risk of bias (**Supplementary Figure S1**), performing well across all key domains. All 6 included non-randomized studies were judged to have a moderate overall risk of bias (**Supplementary Table S3**). The primary source of bias was the non-randomized allocation of interventions, which may have been influenced by treatment era, physician preference, or patient condition, thus introducing selection bias. In addition, incomplete control for baseline confounding was noted in some studies, where groups differed in tumor type, metastasis status, or comorbidity indices at baseline. The remaining domains were generally at low risk of bias.

### Meta-analysis results

#### Technical and clinical success

Technical success rate was reported in all 8 studies (26–33). Pooled analysis showed no statistically significant difference between the EUS-GJ group and the SGJ group (RR = 0.98, 95% CI [0.96, 1.00], P = 0.08) (**Figure 2A**), with low heterogeneity (I^2^ = 0%). In addition, clinical success rate was reported in 8 studies (26–33), revealing no significant difference between the two groups (RR = 1.08, 95% CI [0.99, 1.18], P = 0.07) (**Figure 2B**), with moderate heterogeneity (I^2^ = 64%).

**Figure 2:**
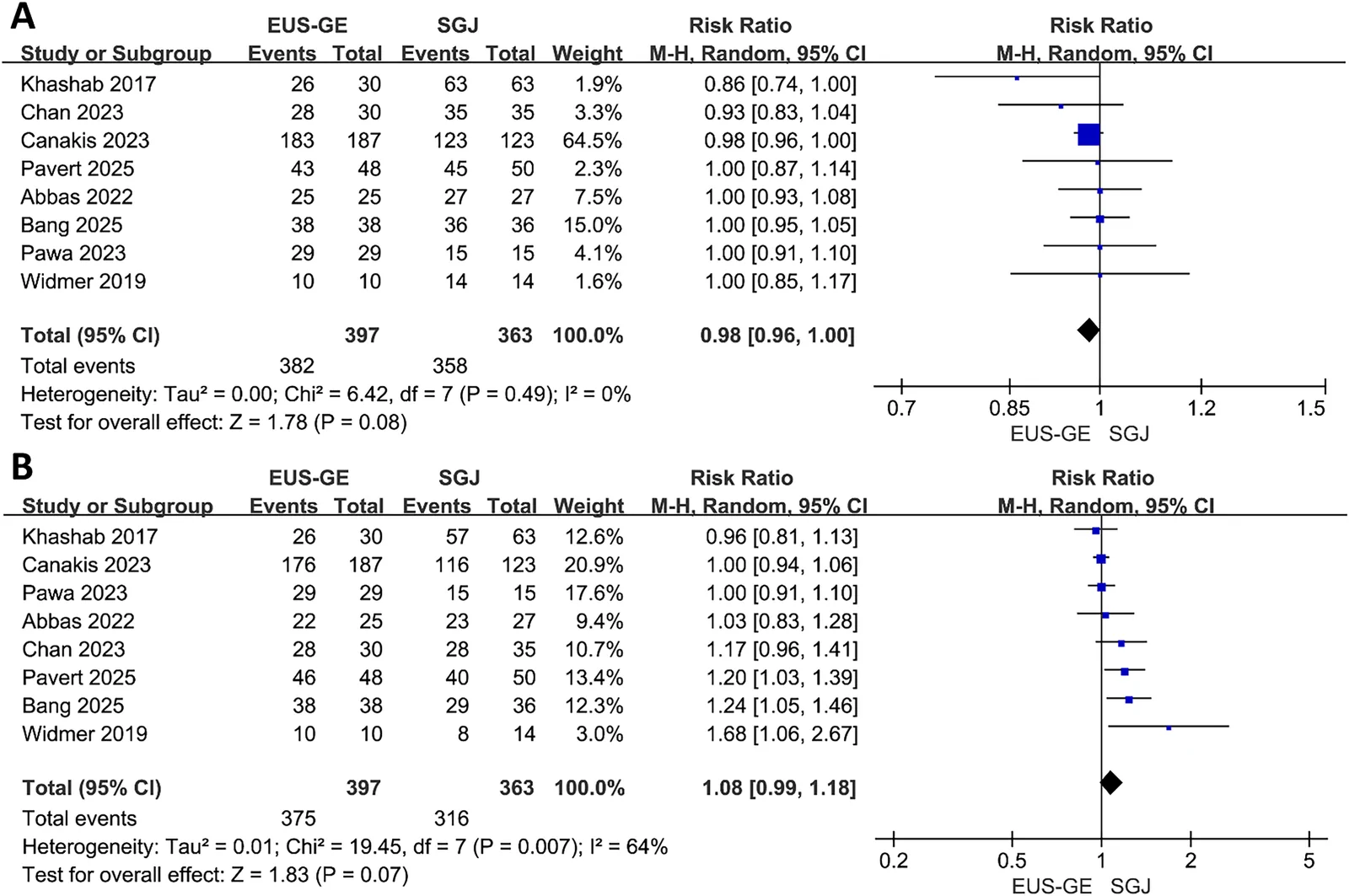
Forest plot comparing technical success rates **(A)** and clinical success rates **(B)** between patients undergoing EUS-GJ and SGJ.

#### Length of hospital stay

Length of hospital stay was reported in 8 studies (26–33). The pooled analysis showed that the postoperative length of hospital stay was significantly shorter in the EUS-GJ group than in the SGJ group (MD = -3.57, 95% CI [-4.76, -2.38], P < 0.001) (**Figure 3**), with high heterogeneity observed by heterogeneity analysis (I^2^ = 85%).

**Figure 3:**
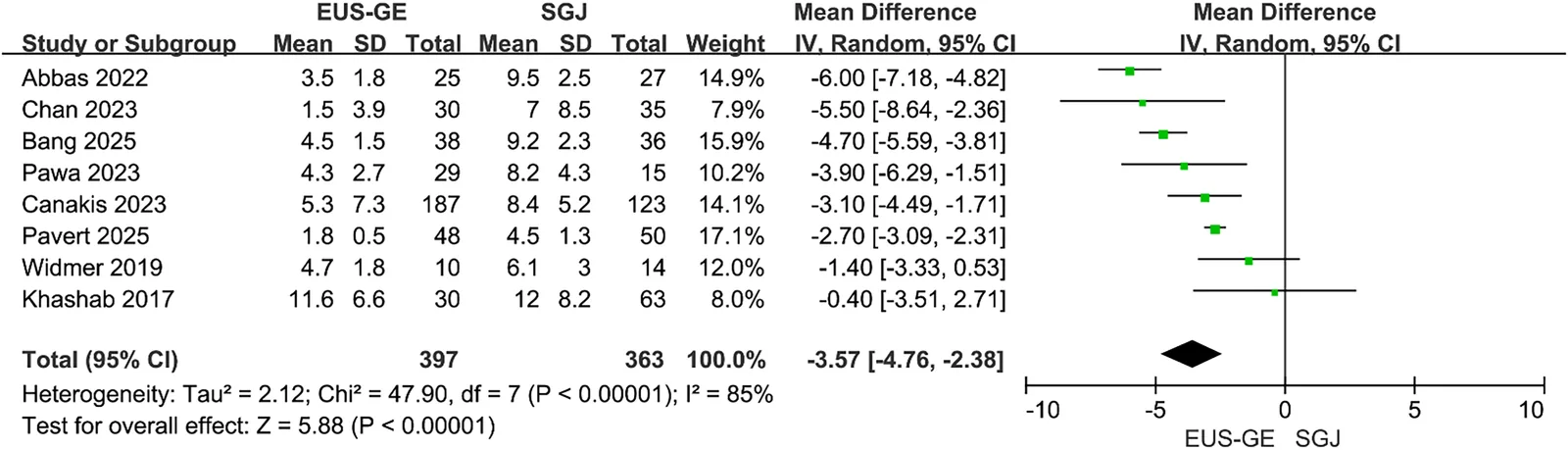
Forest plot comparing length of hospital stay between patients undergoing EUS-GJ and SGJ.

#### Clinical efficacy

Among the clinical efficacy endpoints analyzed, EUS-GJ was associated with a significantly shorter time to first oral intake (MD = -2.88 days, 95% CI [-4.12, -1.01], P < 0.001; 4 studies; I^2^ = 93%) (**Figure 4A**) and a substantially earlier time to resume chemotherapy (MD = -16.05 days, 95% CI [-21.32, -10.78], P < 0.001; 2 studies; I^2^ = 42%) (**Figure 4B**) compared with SGJ. However, no significant differences were observed between the two groups in the proportion of patients tolerating a solid diet at discharge (RR = 1.25, 95% CI [0.69, 2.28], P = 0.46; 3 studies; I^2^ = 81%) (**Figure 4C**), the rate of obstruction recurrence during follow-up (RR = 0.48, 95% CI [0.23, 1.00], P = 0.05; 7 studies; I^2^ = 33%) (**Figure 4D**), or the re-intervention rate (RR = 1.48, 95% CI [0.32, 6.84], P = 0.61; 5 studies; I^2^ = 74%) (**Figure 4E**).

**Figure 4:**
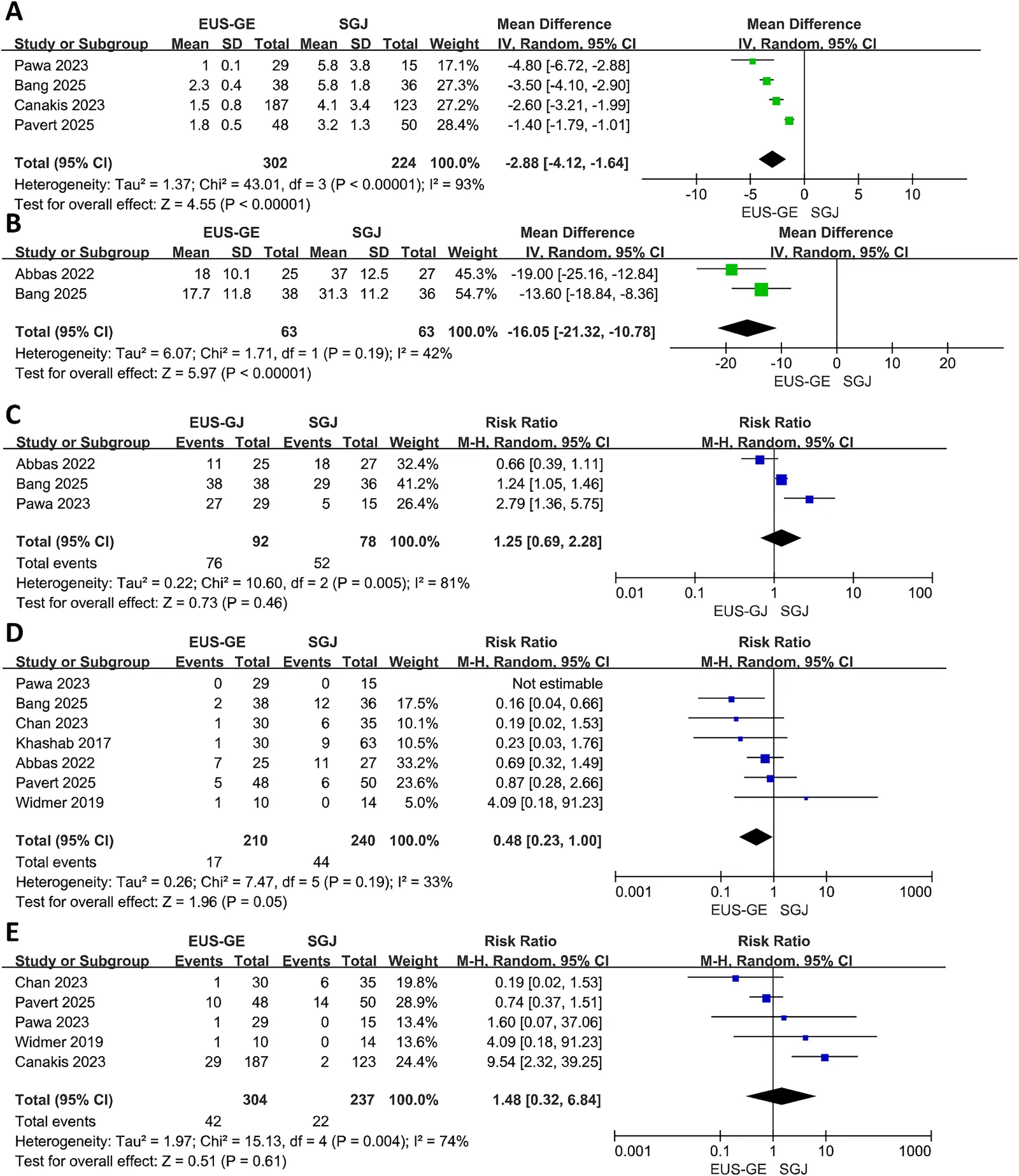
Forest plot comparing clinical efficacy between patients undergoing EUS-GJ and SGJ. **(A)** Forest plot of time to first oral intake; **(B)** Forest plot of time to resume chemotherapy; **(C)** Forest plot of proportion of patients tolerating a solid diet; **(D)** Forest plot of obstruction recurrence; **(E)** Forest plot of re-intervention;.

#### Clinical safety

All 8 studies (26–33) reported overall adverse events. Pooled analysis showed that the EUS-GJ group had a significantly lower overall adverse events rate (RR = 0.44, 95% CI [0.24, 0.81], P = 0.008) (**Figure 5A**), with moderate heterogeneity (I^2^ = 64%). Three studies (26–28) reported 30-day mortality. No significant difference was observed between groups (RR = 1.15, 95% CI [0.44, 3.03], P = 0.77) (**Figure 5B**), with low heterogeneity (I^2^ = 0%).

**Figure 5:**
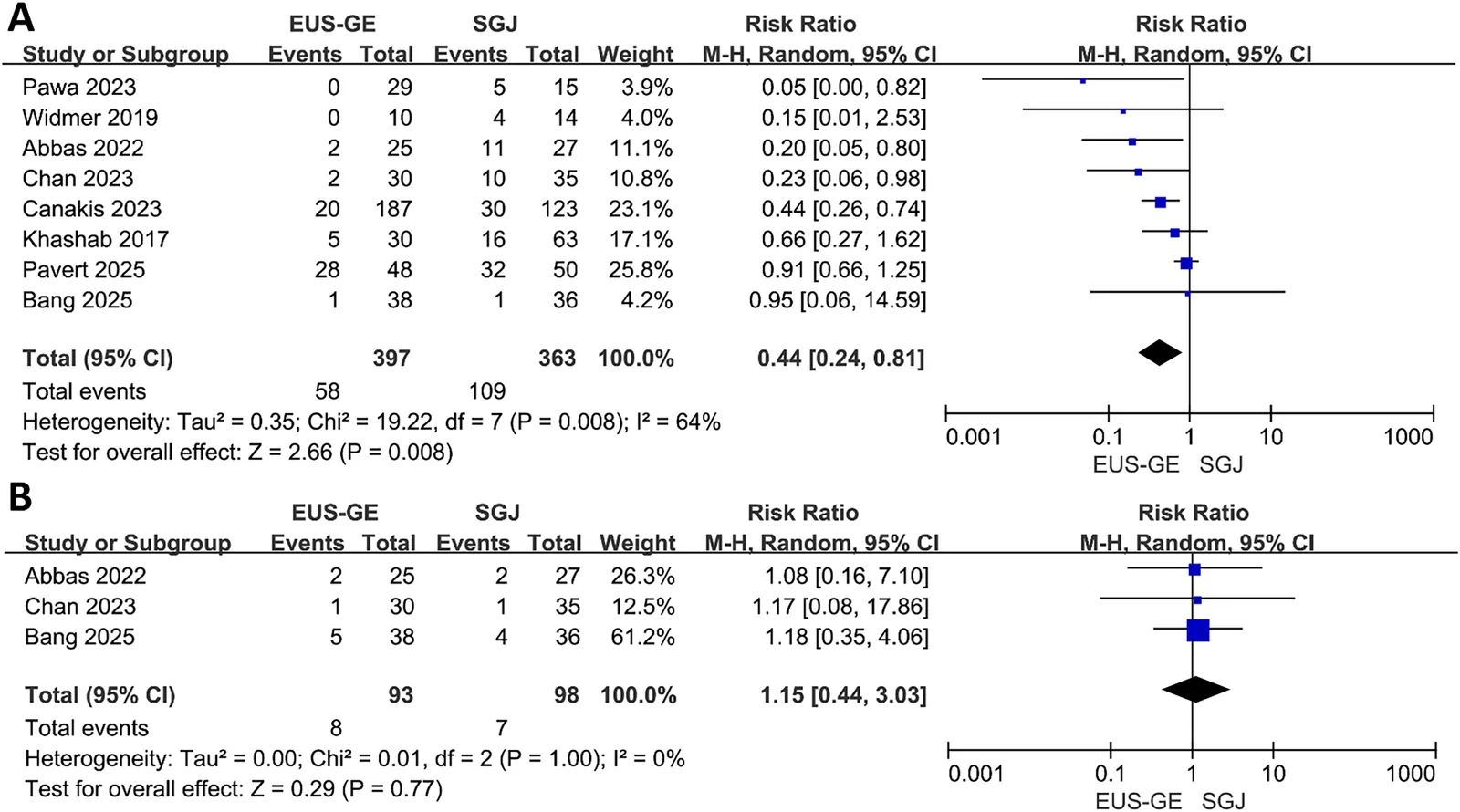
Forest plot comparing clinical safety between patients undergoing EUS-GJ and SGJ. **(A)** Forest plot of overall adverse events; **(B)** Forest plot of 30-day mortality.

#### Subgroup analysis

Subgroup and sensitivity analyses were performed for the five primary outcomes: technical success, clinical success, hospital stay, obstruction recurrence, and overall adverse events. Subgroup analysis according to study design (RCT vs. N-RCT) showed that, within the two RCTs, EUS-GJ was associated with significantly higher clinical success compared with SGJ (RR = 1.22, 95% CI 1.09–1.36, P<0.001), whereas the difference in overall adverse events was not statistically significant (RR = 0.91, 95% CI 0.67–1.25, P = 0.57) (**Supplementary Table S4**). The direction of the pooled estimates for length of hospital stay and obstruction recurrence in the RCT subgroup was consistent with the overall results. The marginal difference in technical success observed in the overall analysis was not maintained in either study-design subgroup, which was consistent with the sensitivity analysis.

Subgroup analysis based on SGJ surgical approach (minimally invasive vs. open) yielded results consistent with the overall pooled estimates for clinical success, length of hospital stay, and obstruction recurrence. Notably, the statistically significant difference in technical success observed in the overall analysis was not reproduced in either subgroup. For overall adverse events, no significant difference was detected between EUS-GJ and the minimally invasive SGJ subgroup (RR = 0.40, 95% CI [0.10, 1.58], P = 0.19), whereas the open SGJ subgroup had a significantly higher adverse event rate than EUS-GJ (RR = 0.43, 95% CI [0.28, 0.66], P < 0.001) (**Supplementary Table S4**).

#### Sensitivity analysis

After omitting the study by Khashab et al. (29) from the analysis, the pooled effect for technical success was attenuated and no longer statistically significant (RR = 0.98, 95% CI [0.95, 1.01], P = 0.22) (**Supplementary Figure S2**), suggesting poor stability of this finding. All other outcomes remained stable in sensitivity analyses.

#### GRADE assessment for primary outcomes

The certainty of evidence for each primary outcome was assessed using the GRADE framework (**Supplementary Table S5**). The certainty of evidence was low for technical success, clinical success, and overall adverse events, downgraded due to risk of bias and imprecision. For hospital stay and obstruction recurrence, the certainty was very low, downgraded for serious inconsistency (hospital stay) and indirectness due to variable follow-up durations (obstruction recurrence), in addition to risk of bias and imprecision. No upgrading factors were identified.

#### Publication bias

Publication bias was assessed using funnel plot visualization and Egger’s test for the five primary outcomes. **Supplementary Figure S3** shows funnel plots for technical success, clinical success, length of hospital stay, obstruction recurrence, and overall adverse events. The funnel plots were generally symmetric, and Egger’s tests were non-significant for all five outcomes, suggesting no evident publication bias.

## Discussion

This systematic review and meta-analysis comprehensively compared the efficacy and safety of EUS-GJ and SGJ in treating MGOO, including 8 clinical studies with 760 patients. The GRADE certainty of evidence for the primary outcomes was low to very low, indicating that the pooled estimates should be interpreted with caution and precluding claims of high-level evidence.

The analysis demonstrated no statistically significant differences between EUS-GJ and SGJ in clinical success (RR = 1.08, P = 0.07), obstruction recurrence (RR = 0.48, P = 0.05), or re-intervention rates (RR = 1.48, P = 0.61). The present analysis detected no statistically significant difference in core efficacy between EUS-GJ and traditional surgery for palliative treatment of MGOO, which aligns with previous findings (17, 20, 34). Both techniques fundamentally aim to restore nutritional intake by creating a gastric bypass, albeit via different routes. Notably, subgroup analysis revealed significantly higher clinical success for EUS-GJ in RCTs (RR = 1.22, < 0.001). This finding is plausible. The multicenter RCT by Pavert et al. (31) reported a clinical success rate of 96% for EUS-GJ versus 80% for SGJ, potentially attributable to standardized procedures (e.g., uniform use of 20mm electrocautery-enhanced LAMS) and balanced baseline characteristics in RCTs, mitigating selection biases common in retrospective studies where EUS-GJ might be preferentially chosen for sicker patients (e.g., higher comorbidity scores, lower BMI), potentially obscuring its inherent efficacy advantage. Taken together, the two included RCTs provide the most methodologically robust evidence and demonstrated that EUS-GJ achieved significantly higher clinical success than SGJ while showing a comparable overall adverse event profile. This suggests that, when selection bias is minimized, EUS-GJ may offer at least equivalent—and possibly superior—efficacy relative to SGJ in appropriately selected patients. However, given the generally low certainty of the evidence, these findings require confirmation in future prospective studies.

The pooled analysis for technical success rate showed a risk ratio of 0.98 (95% CI: 0.96-1.00, P = 0.08). Although this difference did not reach statistical significance, the RR was extremely close to 1 and the upper limit of the confidence interval reached 1.00, indicating that the technical success rates of EUS-GJ and SGJ were essentially equivalent. The minor difference is not clinically significant. This difference can be reasonably explained by the significant learning curve associated with EUS-GJ. The theory proposed by Jovani et al. (35) suggests that approximately 25 procedures are required for an operator to achieve proficiency and at least 40 for mastery, a conclusion supported by subsequent studies (36, 37). Consistent with the learning curve theory, the marginally lower technical success rate of EUS-GJ observed in the early study by Khashab et al. (29) reflects typical limitations during its initial adoption phase, including limited operator experience and evolving procedural standardization (17, 38). Indeed, after omitting this study, the pooled RR changed to 0.98 (95% CI 0.95–1.01, P = 0.22), suggesting that the initially observed marginal difference was unstable and largely driven by a single early experience. With subsequent widespread clinical implementation, accumulated operator expertise, and optimized techniques and devices, the technical success of EUS-GJ has increased significantly. Recent evidence from high-volume centers, such as the study by Abbas et al. (26), confirms a 100% success rate, equivalent to that of SGJ, demonstrating that the marginal difference observed in the pooled analysis is not an inherent limitation of EUS-GJ but a transient outcome influenced by its developmental stage and operator proficiency (39, 40).

The significant advantages of EUS-GJ in recovery-related outcomes represent its core competitive edge. This meta-analysis showed EUS-GJ was associated with a 3.57-day reduction in hospital stay (P<0.001), a 2.88-day earlier resumption of oral intake (P<0.001), and a 16.05-day faster time to chemotherapy resumption (P<0.001). These benefits are inherent to its minimally invasive nature. EUS-GJ is performed endoscopically through the digestive tract, avoiding the trauma of abdominal penetration and tissue separation in open or laparoscopic surgery, and significantly reducing complications that delay recovery such as postoperative pain, paralytic ileus and incisional infection (29, 30, 41). For MGOO patients, most of whom are in the advanced stage of tumors with a median survival of only 3–6 months (42), rapid resumption of oral nutrition can improve malnutrition (2), and early initiation of comprehensive treatments such as chemotherapy and targeted therapy is the key to prolonging survival (33). The recovery advantage of EUS-GJ makes it more suitable for patients who need to initiate tumor comprehensive treatment as soon as possible. Nevertheless, this advantage should be weighed cautiously, as data on time to chemotherapy resumption were derived from only two retrospective studies.

The substantial safety advantage of EUS-GJ demonstrated in this study is an important support for it to become a preferred treatment option. Pooled analysis showed that the incidence of overall adverse events in the EUS-GJ group was only 44% of that in the SGJ group (RR = 0.44, P = 0.008), mainly because EUS-GJ avoids severe complications related to surgery such as infection, anastomotic leakage and postoperative intestinal obstruction. Although EUS-GJ has specific risks such as stent misplacement and puncture-related bleeding, the incidence is low and manageable. Li et al. (20) reported that the incidence of stent misplacement was 5.1%, bleeding 3.1%, and perforation 2.6%, and 89% of stent misplacement could be corrected by endoscopic treatment, with only 11% requiring surgical intervention. A multicenter study by Ghandour et al. (43) further confirmed that among the specific complications of EUS-GJ, mild complications accounted for 60.9% and severe or fatal complications only 15.2%, and the incidence of complications could be reduced from 22% in the early stage to 4% in the later stage with the accumulation of operator experience (39).

Our novel subgroup analysis stratifying SGJ into open versus minimally invasive subgroups helps contextualize previously mixed comparative results from earlier meta-analysis. Prior syntheses aggregated open, laparoscopic and robotic SGJ cases together, which masked heterogeneity within the surgical comparator arm. The subgroup analysis showed no significant difference in adverse events between EUS-GJ and minimally invasive (laparoscopic/robotic) SGJ (RR = 0.40, 95% CI 0.10–1.58, P = 0.19), whereas open SGJ had a significantly higher adverse event rate than EUS-GJ (RR = 0.43, 95% CI 0.28–0.66, P < 0.001), suggesting that the excess safety risk of SGJ mainly stems from trauma-related risks associated with open surgery. The study by Abbas et al. (26) supported this point by reporting that the infection rate of open SGJ was as high as 30%, versus only 10% for laparoscopic SGJ, and even laparoscopic SGJ still had a higher adverse event rate than EUS-GJ (8%). Collectively, these data suggest that although the safety gap between EUS-GJ and SGJ narrows when only laparoscopic/robotic SGJ are considered, EUS-GJ still maintains benefits in hospital stay and time to oral intake, which carries practical clinical meaning: treatment selection should consider not only EUS-GJ operator expertise, but also whether the surgical alternative is open or minimally invasive gastrojejunostomy.

This meta-analysis has several limitations. First, the small number of included studies (two RCTs and six retrospective cohorts) limited statistical power and precluded reliable assessment of publication bias. Second, the exclusion of Pinnam et al. (16), a large nationwide cohort, owing to unavailable patient-level outcomes, may have introduced selection bias and restricted generalizability. Third, the GRADE certainty of evidence was low to very low for all primary outcomes, underscoring the need for cautious interpretation. Fourth, heterogeneity in follow-up durations, sample sizes, baseline characteristics, procedural techniques, and operator experience—along with median-to-mean conversion and variable follow-up periods—may have biased the pooled estimates and underdetected late outcomes. Fifth, the predominantly observational design, including nonconcurrent treatment periods and marked differences in patient fitness, treatment era, institutional expertise, and surgical approach, rendered results susceptible to selection bias and residual confounding. Although sensitivity analyses partially mitigated these concerns, these factors should be considered when interpreting the overall pooled estimates. Finally, because the analyses relied on aggregate data, we could not evaluate baseline contraindications such as ascites or portal hypertension; such patients may have been preferentially treated surgically, potentially confounding the comparative outcomes. Individual patient-level data and prospective registries are needed to confirm our findings.

## Conclusion

This meta-analysis indicates that no statistically significant difference was detected in core efficacy outcomes between EUS-GJ and SGJ for the palliative management of malignant gastric outlet obstruction. Compared with SGJ, EUS-GJ was associated with a shorter hospital stay, earlier resumption of oral intake, earlier resumption of chemotherapy, and a lower risk of overall adverse events. Clinical decision-making should be individualized based on patient anatomy, surgical fitness, life expectancy, and institutional expertise. Given the limitations of the available evidence, future large-scale randomized controlled trials and prospective real-world studies are warranted to confirm the long-term efficacy and broader applicability of EUS-GJ.

## Data Availability

The original contributions presented in the study are included in the article/**Supplementary Material**. Further inquiries can be directed to the corresponding authors.
