# Supplementary material for: Efficacy of endoscopic ultrasound-guided versus surgical gastrojejunostomy for malignant gastric outlet obstruction: a systematic review and meta-analysis
Source: Front Oncol. 2026 Oct 9;16:1943827. doi: 10.3389/fonc.2026.1943827 (PMC13690527; doi:10.3389/fonc.2026.1943827)
Supplement: Supplementary file 1 [file Table1.docx]

**Efficacy of Endoscopic Ultrasound-Guided versus Surgical Gastrojejunostomy for Malignant Gastric Outlet Obstruction: A Systematic Review and Meta-Analysis**

Supplemental Table S1. Detailed search strategies for each database.

Supplementary Table S2. Definitions for Key Outcomes Across Included Studies

Supplementary Table S3. Risk of bias for the included studies, based on the ROBINS-I tool.

Supplementary Table S4. Summary of subgroup analyses.

Supplementary Table S5. GRADE certainty of evidence for primary outcomes.

Supplementary Figure S1. Risk of bias assessment using the Cochrane Risk of Bias Tool version 2.

Supplementary Figure S2. Sensitivity analysis of the technical success.

Supplementary Figure S3. Funnel plot and Egger test results. (A) technical success; (B) clinical success; (C) length of hospital stay; (D) obstruction recurrence; (E) overall adverse events.

Supplemental Table S1. Detailed search strategies for each database.

| Database | Search strategy |
| --- | --- |
| PubMed | #1 (("endoscopic ultrasound-guided"[TI/AB] OR "EUS-guided"[TI/AB] OR "EUS"[TI/AB]) AND ("gastroenterostomy"[TI/AB] OR "gastrojejunostomy"[TI/AB] OR "GE"[TI/AB])) OR ("surgical"[TI/AB] OR "operative"[TI/AB] OR "laparoscopic"[TI/AB] OR "open"[TI/AB]) AND ("gastrojejunostomy"[TI/AB] OR "SGJ"[TI/AB]))  #2 ("malignant"[TI/AB] OR "neoplasm*"[TI/AB] OR "cancer"[TI/AB] OR "carcinoma"[TI/AB] OR "adenocarcinoma"[TI/AB] OR "metastasis"[TI/AB] OR "secondary"[TI/AB])  #3 ("gastric outlet obstruction"[TI/AB] OR "GOO"[TI/AB] OR "pyloric obstruction"[TI/AB] OR "duodenal obstruction"[TI/AB] OR "gastric outlet stenosis"[TI/AB] OR "pyloric stenosis"[TI/AB] OR "gastric outlet blockage"[TI/AB])  #4 #1 and #2 and #3 |
| Embase | 1. exp Gastroenterostomy/  2. exp Gastrojejunostomy/  3. (endoscopic ultrasound guided or EUS-GE or EUS guided).tw,kf.  4. (gastroenterostomy or gastrojejunostomy).tw,kf.  5. 3 and 4  6. 1 or 2 or 5  7. (surgical or operative or laparoscopic).tw,kf.  8. (gastrojejunostomy or SGJ).tw,kf.  9. 7 and 8  10. 6 or 9  11. exp Stomach Neoplasm/ or exp Duodenal Neoplasm/ or exp Pyloric Stenosis/  12. (malignant or cancer* or carcinoma* or adenocarcinoma* or neoplasm*).tw,kf.  13. (gastric outlet obstruction or GOO or pyloric obstruction or duodenal obstruction or gastric outlet stenosis or pyloric stenosis).tw,kf.  14. 12 and 13  15. 11 or 14 |
| Web of Science | TS=((("endoscopic ultrasound" NEAR/2 "gastroenterostomy") OR "EUS-GE" OR ("EUS" NEAR/2 "gastrojejunostomy")) OR (("surgical" OR "laparoscopic") NEAR/2 "gastrojejunostomy") OR "SGJ")) and TS=(("malignant" OR "neoplasm*") NEAR/3 ("gastric outlet obstruction" OR "GOO" OR "pyloric stenosis")) |
| Cochrane Library | #1 (endoscopic ultrasound-guided OR EUS-GE OR EUS-guided) AND (gastroenterostomy OR gastrojejunostomy)  #2 (surgical OR operative OR laparoscopic) AND (gastrojejunostomy OR SGJ)  #3 #1 OR #2  #4 (malignant OR cancer* OR carcinoma* OR neoplasm*) AND ("gastric outlet obstruction" OR GOO OR "pyloric obstruction" OR "duodenal obstruction")  #5 #3 AND #4 |

| Supplementary Table S2. Definitions for Key Outcomes Across Included Studies | | | | | |
| --- | --- | --- | --- | --- | --- |
| Study | Adverse Events (AE) | Recurrence / Re-obstruction | Re-intervention | Oral Intake | Chemotherapy Resumption |
| Khashab 2017 | AEs graded using ASGE lexicon; specific AEs (infection, perforation, bleeding, leak, death) reported. | Recurrent GOO = recurrence of symptoms (nausea, vomiting) requiring intervention (EUS-GE 3% vs SGJ 14%). Mean time to reintervention 88 vs 121 days (NS). | Repeat endoscopic or surgical procedure for recurrent GOO. | Ability to tolerate oral intake without vomiting | Not reported. |
| Widmer 2019 | AEs listed descriptively (e.g., NSTEMI, pneumonia, fistula, haematoma); no uniform grading scale. | Recurrence not separately defined; one food-impaction case in EUS-GE group required reintervention. | Described at case level as endoscopic/surgical management. | GOOSS >=2 (full liquid/soft diet) (EGJ 100% vs SGJ 57%). Assessed at discharge/short-term. | Not reported. |
| Abbas 2022 | AEs graded using ASGE lexicon; 30-day and total follow-up AEs reported (EUS-GE 8% vs SGJ 41%). | Recurrent vomiting/inability to tolerate oral intake within 30 days or follow-up (EUS-GE 28% vs SGJ 41%, NS). | Treatment for obstruction or complications; no separate interval reported. | ability to tolerate oral intake for 30 days post-procedure without obstruction; | Time from procedure to resumption/start of chemotherapy |
| Chan 2023 | 30-day AEs and mortality; classified by standard endoscopic/surgical complication categories, no single severity scale. | Symptom recurrence with endoscopic/radiological confirmation (EPASS 3.3%, LGJ 17.1%, SEMS 36.7%). | Repeat endoscopic or surgical procedure for obstruction or stent dysfunction (EPASS 3.3%, LGJ 17.1%, SEMS 26.5%). | GOOSS improvement >=1; 1-month GOOSS reported; | Not reported. |
| Canakis 2023 | AEs graded by Clavien-Dindo; total AE rates reported overall and by laparoscopic-surgery subgroup. | Recurrence not separately defined; reintervention used as proximal indicator. | Not reported. | Time to resumption of oral intake in days; clinical success = tolerance of at least full-liquid diet. No uniform GOOSS use. | Not reported. |
| Pawa 2023 | AEs graded using ASGE lexicon (EUS-GE 0/29 vs R-GJ 5/15). Window: hospitalisation and >=1-month follow-up. | No separate recurrence rate; GOOSS at 1 month used as proxy. | Reintervention reported at case level. | Tolerance of oral intake at hospital discharge; 1-month GOOSS reported. | Not reported. |
| Pavert 2025 | Overall AEs, serious AEs and death reported separately; no single grading system. | Persistent or recurrent obstructive symptoms requiring reintervention was the second coprimary outcome (non-inferiority margin 20%). | Any endoscopic, surgical or radiological intervention required for obstructive symptoms. | GOOSS >=2 without vomiting (median 1 vs 3 days). Assessed daily during admission and follow-up. | Time from procedure to resumption/start of chemotherapy |
| Bang 2025 | Procedure-related AEs graded by Clavien-Dindo; disease-related AEs reported separately. | Persistent/recurrent obstruction captured within the composite endpoint (GOOSS 0/1 at discharge, need for reintervention or supplemental nutrition). | Additional endoscopic or surgical intervention or supplemental nutrition (e.g., feeding tube, parenteral nutrition). | Oral intake assessed using GOOSS; time to solid diet = GOOSS >=2 (median 2 vs 5 days, EUS-GE vs SGJ). Assessed at discharge and up to 6 months. | Not a prespecified outcome for pooling. |

EUS-GE, endoscopic ultrasound-guided gastroenterostomy; SGJ, surgical gastrojejunostomy; GOOSS, Gastric Outlet Obstruction Scoring System; AE, adverse event; RCT, randomised controlled trial.

| Supplementary Table S3 Risk of bias for the included studies, based on the ROBINS-I tool. | | | | | | | | | |
| --- | --- | --- | --- | --- | --- | --- | --- | --- | --- |
| Author | Year | Type of bias | | | | | | | Overall rating |
|  |  | Confounding | Selection of participants | Measurement classification of interventions | Deviations from intended interventions | Missing data | Measurement of the outcomes | Selection of the reported result |  |
| Khashab | 2017 | Moderate | Moderate | Low | Low | Low | Low | Low | Moderate |
| Widmer | 2019 | Moderate | Moderate | Low | Low | Low | Low | Low | Moderate |
| Abbas | 2022 | Low | Moderate | Low | Low | Low | Low | Low | Moderate |
| Chan | 2023 | Moderate | Moderate | Low | Low | Low | Low | Low | Moderate |
| Pawa | 2023 | Low | Moderate | Low | Low | Low | Low | Low | Moderate |
| Canakis | 2023 | Moderate | Moderate | Low | Low | Low | Low | Low | Moderate |

ROBINS-I: risk of bias in non-randomized studies of interventions; NA=not applicable

| Supplementary Table S4 Summary of subgroup analyses. | | | | | | |
| --- | --- | --- | --- | --- | --- | --- |
| Primary Outcomes | Subgroup | Number | MD or RR | 95%CI (lower, upper) | I^2^ | P-value |
| Technical success | Study design |  |  |  |  |  |
|  | N-RCT | 6 | 0.98 | (0.94, 1.01) | 13% | 0.13 |
|  | RCT | 2 | 1.00 | (0.95, 1.05) | 0% | 0.98 |
|  | Surgical methods |  |  |  |  |  |
|  | O-SGJ | 4 | 0.97 | (0.92, 1.03) | 46% | 0.34 |
|  | M-SGJ | 4 | 0.98 | (0.93, 1.04) | 0% | 0.52 |
| Clinical success | Study design |  |  |  |  |  |
|  | N-RCT | 6 | 1.08 | (0.97, 1.20) | 62% | 0.15 |
|  | RCT | 2 | 1.22 | (1.09, 1.36) | 0% | < 0.001 |
|  | Surgical methods | | | | | |
|  | O-SGJ | 4 | 1.02 | (0.91, 1.15) | 47% | 0.70 |
|  | M-SGJ | 4 | 1.14 | (0.99, 1.30) | 69% | 0.06 |
| Length of stay | Study design | | | | | |
|  | N-RCT | 6 | -3.27 | (-4.77, -1.76) | 86% | < 0.001 |
|  | RCT | 2 | -3.66 | (-5.62, -1.70) | 94% | < 0.001 |
|  | Surgical methods | | | | | |
|  | O-SGJ | 4 | -2.94 | (-5.39, -0.48) | 88% | 0.02 |
|  | M-SGJ | 4 | -3.96 | (-5.46, -2.46) | 84% | < 0.001 |
| Recurrence of obstruction | Study design | | | | | |
|  | N-RCT | 5 | 0.64 | (0.36, 1.15) | 1% | 0.14 |
|  | RCT | 2 | 0.39 | (0.07, 2.14) | 72% | 0.28 |
|  | Surgical methods | | | | | |
|  | O-SGJ | 3 | 0.65 | (0.25, 1.69) | 16% | 0.38 |
|  | M-SGJ | 4 | 0.34 | (0.10, 1.16) | 51% | 0.08 |
| Overall adverse event | Study design | | | | | |
|  | N-RCT | 6 | 0.48 | (0.26, 0.86) | 66% | 0.01 |
|  | RCT | 2 | 0.91 | (0.67, 1.25) | 0% | 0.57 |
|  | Surgical methods | | | | | |
|  | O-SGJ | 4 | 0.43 | (0.28, 0.66) | 0% | < 0.001 |
|  | M-SGJ | 4 | 0.40 | (0.10, 1.58) | 68% | 0.19 |

RCT: randomized controlled trial; O-SGJ: open surgical gastrojejunostomy; M-SGJ: minimally invasive surgical gastrojejunostomy;

| Supplementary Table S5 GRADE certainty of evidence for primary outcomes. | | | | | | |
| --- | --- | --- | --- | --- | --- | --- |
| Outcomes | **Anticipated absolute effects^*^** (95% CI) | | Relative effect (95% CI) | № of participants (studies) | Certainty of the evidence (GRADE) |  |
|  | **Risk with SGJ** | **Risk with EUS-GJ** |  |  |  |  |
| Technical success | 986 per 1,000 | **967 per 1,000** (947 to 986) | **RR 0.98** (0.96 to 1.00) | 760 (8 non-randomised studies) | ⨁⨁◯◯ Low |  |
| Clinical success | 871 per 1,000 | **940 per 1,000** (862 to 1,000) | **RR 1.08** (0.99 to 1.18) | 760 (8 non-randomised studies) | ⨁⨁◯◯ Low |  |
| Obstruction recurrence | 183 per 1,000 | **88 per 1,000** (42 to 183) | **RR 0.48** (0.23 to 1.00) | 450 (7 non-randomised studies) | ⨁◯◯◯ Very low |  |
| Length of hospital stay | 0 | MD **3.57 lower** (4.76 lower to 2.38 lower) | - | 760 (8 non-randomised studies) | ⨁◯◯◯ Very low |  |
| Overall adverse eventss | 300 per 1,000 | **132 per 1,000** (72 to 243) | **RR 0.44** (0.24 to 0.81) | 760 (8 non-randomised studies) | ⨁⨁◯◯ Low |  |
| ***The risk in the intervention group** (and its 95% confidence interval) is based on the assumed risk in the comparison group and the **relative effect** of the intervention (and its 95% CI). **CI:** confidence interval; **MD:** mean difference; **RR:** risk ratio | | | | | | |

Supplementary Figure S1. Risk of bias assessment using the Cochrane Risk of Bias Tool version 2.

Supplementary Figure S2. Sensitivity analysis of the technical success.

Supplementary Figure S3. Funnel plot and Egger test results. (A) technical success; (B) clinical success; (C) length of hospital stay; (D) obstruction recurrence; (E) overall adverse events.
